# Risk factors for complications in patients undergoing gastrointestinal endoscopy under acupuncture anesthesia

**DOI:** 10.12669/pjms.40.9.10087

**Published:** 2024-10

**Authors:** Haoliang Cai, Xiaohui Wu, Xi Chen, Jun Guo, Wenting Chen

**Affiliations:** 1 Haoliang Cai, Department of Anesthesiology, Shuguang Hospital, Affiliated to Shanghai University of Traditional Chinese Medicine, Shanghai, P.R. China; 2 Xiaohui Wu, Department of Anesthesiology, Shuguang Hospital, Affiliated to Shanghai University of Traditional Chinese Medicine, Shanghai, P.R. China; 3 Xi Chen, Department of Anesthesiology, Shuguang Hospital, Affiliated to Shanghai University of Traditional Chinese Medicine, Shanghai, P.R. China; 4 Jun Guo, Department of Anesthesiology, Shuguang Hospital, Affiliated to Shanghai University of Traditional Chinese Medicine, Shanghai, P.R. China; 5 Wenting Chen, Department of Anesthesiology, Shuguang Hospital, Affiliated to Shanghai University of Traditional Chinese Medicine, Shanghai, P.R. China

**Keywords:** Gastrointestinal endoscopy, Acupuncture anesthesia, Complications, Nomogram

## Abstract

**Objective:**

To identify risk factors for complications in patients undergoing gastrointestinal endoscopy under acupuncture anesthesia and to construct a nomogram predictive model.

**Methods:**

This retrospective study included 292 patients who underwent gastrointestinal endoscopy under acupuncture anesthesia at the Shuguang Hospital Affiliated to Shanghai University of Traditional Chinese Medicine from June 2020 to May 2023. Logistic regression analysis was used to identify risk factors for complications in patients undergoing gastrointestinal endoscopy under acupuncture anesthesia. A nomogram prediction model was constructed using the RMS package of R4.1.2 software based on the independent risk factors identified. The predictive performance of the model was assessed using consistency index (C-index), calibration curve, and receiver operating characteristic (ROC) curve.

**Results:**

Seventy-five patients (25.68%) had complications. Body mass index (BMI), history of cardiovascular diseases, fasting time, history of respiratory diseases, and Sedation-Agitation Scale (SAS) score were identified as risk factors for complications. Based on this risk, a nomogram predictive model was constructed. The C-index of the nomogram model was 0.927. Calibration curve showed a good consistency between actual observations and nomogram predictions. The ROC curve area under curve (AUC) was 0.927 (95% CI: 0.895-0.959), indicating a certain predictive value for the occurrence of complications. When the optimal cut-off value was selected, the sensitivity and specificity of the model were 77.0% and 92.0%, respectively, indicating that the predictive model was effective.

**Conclusions:**

BMI, history of cardiovascular disease, fasting time, history of respiratory disease, and SAS score are independent risk factors for complications in patients undergoing gastrointestinal endoscopy under acupuncture anesthesia. The constructed nomogram predictive model has a good performance in predicting the occurrence of complications in patients undergoing gastrointestinal endoscopy with under acupuncture anesthesia.

## INTRODUCTION

Gastrointestinal endoscopy is a common method in clinical diagnosis and treatment of gastrointestinal diseases,^1^ which has the advantages of high diagnostic accuracy, short examination time, and less pain.^1^,^2^ However, gastrointestinal endoscopy is an invasive procedure^3^ that may cause damage to the gastrointestinal mucosa and is associated with some adverse reactions, such as nausea and vomiting, which may require medical intervention in severe cases.^3^,^4^

Acupuncture anesthesia is widely used in gastrointestinal endoscopy as a non-pharmacological anesthesia method,^5^ where injection of local anesthetic drugs is performed to block nerve conduction in specific areas.^6^ This type of anesthesia is characterized by fast onset, and minimal side effects.^5^,^6^ However, studies show that acupuncture anesthesia is still associated with a certain risk of complications, such as trauma to the skin and deep tissues, pain,^7^ local bleeding, which can lead to serious consequences such as hematoma, cardiovascular accidents, respiratory depression, hypotension, etc.^6^-^8^ Although there are numbers of studies on the risk factors for complications of gastrointestinal endoscopy^9^ and the effect of acupuncture in gastrointestinal endoscopy,^10^ few studies have explored the risk factors for complications in patients undergoing gastrointestinal endoscopy under acupuncture anesthesia, let alone contrasting a nomogram predictive model for complications. This study aimed to identify risk factors for complications in patients undergoing gastrointestinal endoscopy with acupuncture anesthesia and construct a nomogram predictive model for complications.

## METHODS

This retrospective study included 292 patients (173 males and 119 females) who underwent gastrointestinal endoscopy with acupuncture anesthesia at the Shuguang Hospital Affiliated to Shanghai University of Traditional Chinese Medicine from June 2020 to May 2023.

### Ethical Approval:

The ethics committee of Shuguang Hospital Affiliated to Shanghai University of Traditional Chinese Medicinel approved this study with the number 2023-1327-94-01 on May 31, 2023.

### Inclusion criteria:

- Patients >18 years old who met the indications for painless gastrointestinal endoscopy.
- Patients with complete clinical data.

### Exclusion criteria:

- Patients with comorbid mental illness.
- Patients with severe cardiovascular disease.
- Patients who had undergone invasive gastrointestinal examinations in the past six months or with a history of gastrointestinal resection.
- Patients with combined volvulus or intussusception.

### Acupuncture anesthesia:

Emergency equipment, monitors, oxygen inhalation equipment, medication were prepared before the examination. Patients fasted for eight hours prior to the procedure. For patients undergoing colonoscopy, intestinal preparation was done. Acupuncture anesthesia was initiated 20 minutes before the examination. Patients were instructed to take a sitting position, and routine disinfection was performed. A millineedle (0.30mm) × 40mm needling was used for the needling of the ear acupoint Shenmen, shallow needling 1mm before fixation. Bilateral Neiguan and Hegu acupoints were acupunctured at a depth of 5-10mm. A needle was left for 20 minutes before checking again. Patient was instructed to take the left lying position during the examination. A venous channel was established, the patient was connected to the electrocardiogram monitor, and the oxygen flow rate was maintained at 2-4L per minute. Nasal cannula oxygen inhalation was initiated, and anesthesiologist slowly injected 0.05mg fentanyl (manufacturer: National Pharmaceutical Group Industry Co., Ltd; China). After three minutes, 1.0mg/kg propofol (manufacturer: Jiangsu Ehwa Pharmaceutical Co., Ltd; China) was injected separately, with an injection rate of 40-60mg/minutes. At the same time, the electroacupuncture instrument was connected to Neiguan and Hegu acupoints, with a parameter of 1Hz and a constant current output stimulus intensity of 10-20mA, until the examination result was obtained. After the eyelash reflex and consciousness disappeared, a gastroscopy examination was performed. First, the secretions from the throat were aspirated to avoid coughing. When the colonoscope reached the ileocecal valve or the gastroscope reached the descending part of the duodenum, anesthesia infusion was stopped.

During the examination, propofol was selectively added based on the patient’s facial color changes and limb movements, usually adding 10-30mg each time. For patients undergoing combined gastroscopy and colonoscopy examinations, a regular mask was used for oxygen inhalation after the gastroscopy was completed. The same position was maintained, the anesthesia was increased, and the colonoscopy examination was performed. Throughout the entire inspection process, close monitoring of vital signs and timely detection of abnormalities was done.

### Observation indicators:

- Patient gender, age, smoking history, chronic disease, examination method, body mass index (BMI), history of cardiovascular diseases, fasting time, history of respiratory diseases, Self-Rating Anxiety Scale (SAS) score (SAS score includes a total of 20 items, with each item scoring 1-4 points. A baseline score of 50 points is used, and a score greater than 50 points indicates the presence of anxiety).^11^
- ***Complications:*** nausea and vomiting, transient hypotension, severe bloating, abdominal pain, and anesthesia related complications (hypoxia, hypotension, gastrointestinal reactions, respiratory depression.

### Statistical analysis:

The statistical software SPSS 26.0 and R software version 4.0.0. were used for analysis. The measurement data were represented as mean ± standard deviation, and independent sample t-test was used for inter-group comparison; The counting data were represented by n (%). Comparison between groups was done using *χ^2^*. Univariate and multivariate logistic regression model were used to analyze the risk factors of complications. A nomogram predictive model was constructed using the “rms” package in R software, and ROC curve analysis was used to predict performance. *P*<0.05 indicated statistically significant differences.

## RESULTS

As shown in Table-I, of 292 patients undergoing gastrointestinal endoscopy with acupuncture anesthesia, 75 (25.68%) had complications. Univariate logistic analysis showed that BMI, history of cardiovascular diseases, fasting time, history of respiratory diseases, and SAS score had an impact on the occurrence of complications in patients undergoing gastrointestinal endoscopy with acupuncture anesthesia (*P*<0.05) (Table-II). The results of the multivariate logistic regression model showed that BMI, history of cardiovascular diseases, fasting time, history of respiratory diseases, and SAS score were independent influencing factors for the occurrence of complications in patients undergoing gastrointestinal endoscopy with acupuncture anesthesia (*P*<0.05) (Table-III).

**Table-I:** Analysis on the composition ratio of complications in patients undergoing gastrointestinal endoscopy under acupuncture anesthesia.

Index	n	Constituent ratio (Total = 292, %)
Nausea and vomiting	21	7.19
Transient hypotension	16	5.48
Severe flatulence	15	5.14
Abdominal pain	13	4.45
Anesthesia-related complications (hypoxia, hypotension, gastrointestinal reactions, respiratory depression, etc.)	10	3.42
Amount to	75	25.68

**Table-II:** Analysis of the risk factors associated with complications in patients undergoing gastrointestinal endoscopy under acupuncture anesthesia.

Index	Classify	A group occurred (n=75)	No group occurred (n=217)	χ^2^/t	P
Gender	Male	45 (60.00)	128 (58.99)	0.024	0.878
	Female	30 (40.00)	89 (41.01)		
Age (years)	/	60.45±6.70	59.19±7.74	1.260	0.209
History of smoking	Yes	50 (66.67)	148 (68.20)	0.060	0.806
	No	25 (33.33)	69 (31.80)		
BMI (kg/m²)	/	24.89±2.94	23.03±2.95	4.724	<0.001
History of cardiovascular system disease	Yes	55 (73.33)	63 (29.03)	45.426	<0.001
	No	20 (26.67)	154 (70.97)		
Chronic disease	Merge	26 (34.67)	79 (36.41)	0.073	0.787
	Not merged	49 (65.33)	138 (63.59)		
Fasting time	/	3.83±1.39	4.49±1.23	-3.689	<0.001
History of respiratory disease	Yes	60 (80.00)	61 (28.11)	61.841	<0.001
	No	15 (20.00)	156 (71.89)		
Test mode	Gastroscope	29 (38.67)	83 (38.25)	0.019	0.991
	Enteroscopy	31 (41.33)	89 (41.01)		
	Gastroenterological endoscope	15 (20.00)	45 (20.74)		
SAS grade	>50grade	57 (76.00)	60 (27.65)	54.260	<0.001
	≤50grade	18 (24.00)	157 (72.35)		

**Table-III:** Independent risk factors associated with the occurrence of complications in patients undergoing gastrointestinal endoscopy under acupuncture anesthesia.

Variable	B	S.E.	Wald	P	OR	95% C.I.
BMI (kg/m^2^)	0.218	0.073	8.915	0.003	0.804	0.697~0.928
History of cardiovascular system disease	2.064	0.415	24.781	<0.001	7.877	3.495~17.754
Fasting time (hour)	-0.386	0.145	7.033	0.008	1.47	1.106~1.955
History of respiratory disease	2.766	0.445	38.703	<0.001	15.888	6.648~37.972
SAS grade (component)	2.333	0.425	30.094	<0.001	10.309	4.479~23.727
Constant (quantity)	-1.216	1.802	0.456	0.5	3.374	

**Fig.1:**
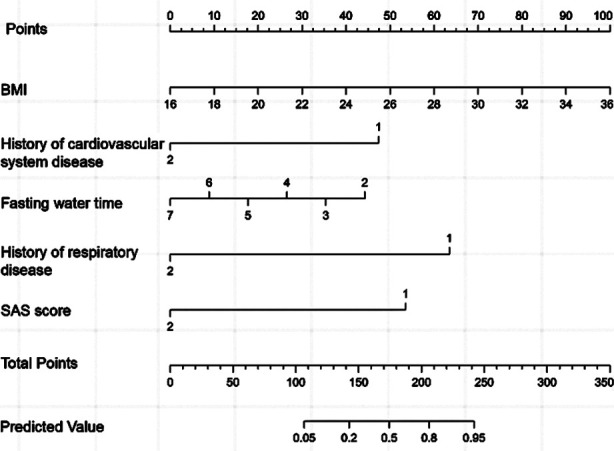
Nomogram predictive model for complications in patients undergoing gastrointestinal endoscopy under acupuncture anesthesia.

**Fig.2:**
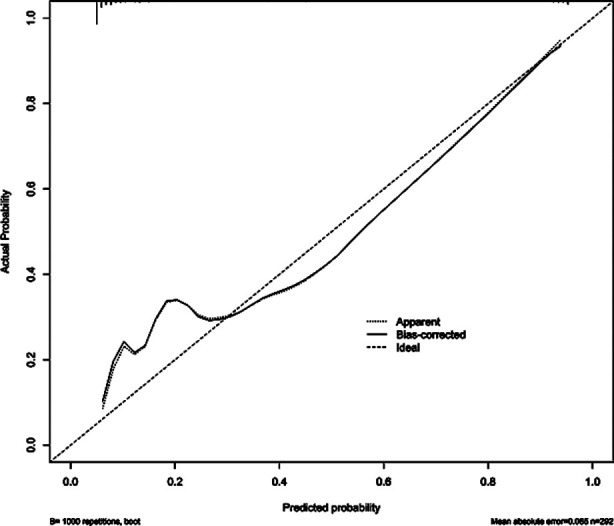
Calibration curve of the nomogram model.

**Fig.3:**
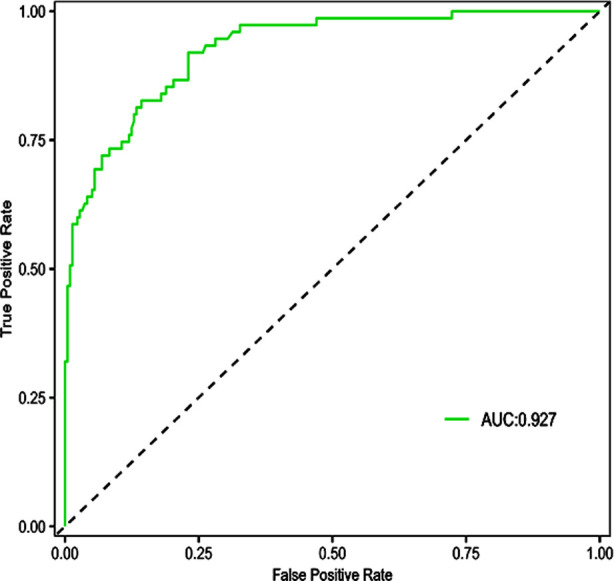
ROC curve.

A nomogram predictive model of significant risk factors was then drawn in a multivariate logistic regression model, including BMI, history of cardiovascular disease, fasting time, history of respiratory disease, and SAS score. The nomogram predictive model predicted the occurrence of complications, and the C-index of the nomogram model was 0.927, reflecting the sufficient discrimination ability of the prediction model (Fig.1). Calibration curve also showed a good consistency between actual observations and nomogram predictions (Fig.2). A ROC curve was established to analyze the value of the model in predicting the occurrence of comorbidities. The AUC was 0.927 (95% CI: 0.895-0.959), with sensitivity of 77.0%, specificity of 92.0%, and the cut-off value of 0.146. These results indicated that the accuracy of the model was good, and it has a good performance in predicting the occurrence of complications in patients undergoing gastrointestinal endoscopy with acupuncture anesthesia (Fig.3).

## DISCUSSION

We noticed that high BMI, history of cardiovascular diseases, short fasting time, history of respiratory diseases, and high SAS score are independent risk factors for the occurrence of complications in patients undergoing gastrointestinal endoscopy under acupuncture anesthesia.

Previous studies have shown the negative impact of weight gain on the cardiovascular system, including a proportional increase in blood volume and cardiac output, leading to increased pre- and post-load on the heart, and an increase in pulmonary blood volume and pulmonary arterial hypertension.^12^,^13^ Additionally, weight gain may lead to imbalance between oxygen supply and consumption.^13^ A study by Kivimak et al.^14^ showed that obesity increases the risk of cardiovascular and nerve diseases, disorder of digestive and respiratory tract, and musculoskeletal disorders. Patients with cardiovascular diseases are more sensitive to stress, associated with acupuncture anesthesia, which in turn affects vital signs and anesthesia effectiveness, thereby increasing the risk of complications.^15^ Additionally, population of patients with cardiovascular diseases has higher incidence of heart failure and myocardial ischemia, which can affect gastrointestinal peristalsis, increase operational difficulty and risk.^16^ These patients also have poor vascular elasticity and are prone to circulatory disorders, leading to tissue hypoxia and acidosis, thereby increasing the risk of complications.^17^

Selection of appropriate type of anesthesia is important for ensuring the safety of the gastrointestinal endoscopy. For example, muscle relaxants often lead to oxygen entry into the stomach, resulting in reduced gastrointestinal motility and decreased gastric tone.^18^,^19^ Inadequate fasting time can cause drug aspiration in the stomach, and higher risk of food and liquid reflux into the esophagus.^19^,^20^ Studies have shown that patients with a history of esophageal stenosis or gastroesophageal reflux disease are especially predisposed to reflux into the respiratory tract, leading to complications such as aspiration pneumonia.^21^

Accurate monitoring of the respiratory status of patients undergoing gastrointestinal endoscopy under acupuncture anesthesia may be challenging, which affect the ability to detect and address potential respiratory problems in a timely manner, increasing the risk of complications.^20^,^21^ In addition, gastrointestinal endoscopy requires injection of short-acting anesthetics. These drugs may cause respiratory suppression, especially for patients with respiratory infections such as colds and bronchitis. In addition, the use of anesthetic drugs can suppress normal breathing, leading to sudden respiratory arrest.^22^,^23^ Research has shown that fear, tension, and anxiety in patients may affect their physiological state, leading to increased blood pressure and heart rate, and increasing the risk of cardiovascular and cerebrovascular diseases.^24^ Negative emotions during gastrointestinal endoscopy may also affect patient’s perception of pain. It can stimulate the sympathetic nervous system, cause coronary artery contraction, promote increased adrenaline secretion, and cause complications such as transient hypotension.^24^,^25^

To reduce the incidence of complications, corresponding intervention guidance needs to be provided for the risk factors in this population of patients. It is suggested that:

1. Thorough evaluation of the patient’s psychological state is required;
2. Preventive measures for possible complications should be prepared in advance;
3. Preoperative fasting period should be at least two hours to reduce the risk of aspiration or suffocation during the examination process
4. Appropriate anesthetic drugs with high safety and minimal side effects should be chosen based on the patient’s specific situation and doctor’s advice;
5. During the procedure, patients should maintain a supine position with their head slightly tilted back, which can effectively maintain airway patency and reduces the risk of respiratory obstruction. In addition, special attention should be paid to the heart rate, blood pressure, blood oxygen saturation and other indicators to avoid cardiovascular and cerebrovascular complications;
6. After the procedure, patients should receive specific nutritional and physical activity instructions. All changes in patients’ condition need to be monitored.

Based on the identified risk factors (BMI, history of cardiovascular disease, fasting time, history of respiratory disease, and SAS score), we constructed and validated a risk prediction model for the occurrence of complications, and found that this model can achieve good prediction results. These indicators are relatively easy to obtain in clinical practice. Therefore, our model can be used to predict the risk of complications in patients who undergoing gastrointestinal endoscopy under acupuncture anesthesia in clinical practice.

### Limitations:

It is a retrospective, single-center study with small sample size, which carries a risk of the selection bias. The repeatability and robustness of the nomogram model need to be validated in prospective multicenter studies with larger datasets. Further expansion of sample size, multicenter research, and inclusion of more relevant factors for analysis are needed to improve the reliability and generalization ability of our conclusions.

## CONCLUSION

High BMI, history of cardiovascular diseases, fasting time, history of respiratory diseases, and high SAS score are risk factors leading to complications in patients undergoing gastrointestinal endoscopy under acupuncture anesthesia. The prediction model constructed in this study has a certain value. Corresponding countermeasures and interventions targeting identified risk factors, and timely evaluation of the patient’s overall condition can ensure smooth examination and reduce the occurrence of complications.

### Authors’ contributions:

**HC:** Conceived and designed the study.

**HC, XW, XC, JG** and **WC:** Collected the data and performed the analysis.

**HC** and **WC:** Was involved in the writing of the manuscript and is responsible for the integrity of the study.

All authors have read, critically reviewed and approved the final manuscript.
